# The Hidden Costs of ICU Caregiving: Economic, Mental Health and Spiritual Consequences

**DOI:** 10.3390/healthcare14040487

**Published:** 2026-02-14

**Authors:** Fotios Tatsis, Mary Gouva, Elena Dragioti, Foteini Veroniki, Konstantinos Stamatis, Georgios Papathanakos, Vasilios Koulouras

**Affiliations:** 1 Intensive Care Unit, University Hospital of Ioannina, 45500 Ioannina, Greece; 2 Scientific Laboratory of Psychology and Person-Centered Care, Department of Nursing, School of Health Sciences, University of Ioannina, 45500 Ioannina, Greecedragioti@uoi.gr (E.D.)

**Keywords:** ICU caregivers, economic burden, psychopathology, spirituality, resilience, coping strategies

## Abstract

**Background**: Family caregivers of intensive care unit (ICU) patients face a double burden: the psychological toll of critical illness and the economic and occupational disruptions that often accompany prolonged caregiving. While prior research has examined caregiver distress, few studies have systematically integrated economic, psychological and spiritual domains over long-term follow-up. **Methods**: This study presents a cross-sectional analysis conducted at long-term follow-up examining economic, occupational, psychological, and spiritual correlates among family caregivers of former ICU patients. From an initial cohort of 189 caregivers, 92 participated in a five-year follow-up, completing validated psychometric instruments (SCL-90-R, SpREUK, CD-RISC-10, Heartland Forgiveness, F-COPES). Multivariate regression models were used to identify predictors of psychological and spiritual outcomes, while cluster analysis explored heterogeneity in caregiver profiles. **Results**: Job loss emerged as a strong predictor of anxiety and hostility, while reduced working hours showed a protective association against depression and anxiety. Financial burden was less consistently associated with psychopathology. Spirituality demonstrated an ambivalent pattern of correlational associations: while dimensions such as trust and reflection were linked to adaptive coping, higher levels of spiritual engagement were also associated with elevated depressive symptoms, suggesting a reactive rather than purely protective role. Resilience and coping resources (e.g., reframing, forgiveness, personal competence) mitigated distress, whereas neuroticism amplified vulnerability. Cluster analysis revealed three distinct caregiver subgroups: a high-burden cluster (severe psychopathology and economic strain), a moderate cluster with mixed spiritual and psychological engagement and a resilient cluster with minimal burden. **Conclusions**: This study highlights that economic stressors are not peripheral but central drivers of caregiver distress and that spirituality, although valued, may operate in both adaptive and maladaptive ways. Tailored interventions must integrate financial protection, psychological support and sensitive spiritual care to address the multidimensional reality of ICU caregiving.

## 1. Introduction

Hospitalization in Intensive Care Units (ICUs) represents one of the most critical and psychologically distressing experiences not only for patients but also for their relatives. The sudden admission, the uncertainty regarding survival and the prolonged length of stay in the ICU generate a complex field of psychological, social and economic consequences. Family members are required not only to assume the role of caregiver during hospitalization but also to adapt to a new reality in which the patient’s health and needs largely dictate daily life. International literature highlights that relatives caring for ICU patients experience significant psychological strain, often accompanied by professional and financial difficulties that may persist long after the patient’s discharge [1,2].

In practice, family caregivers frequently reduce their working hours or temporarily or permanently leave their professional occupations, leading to income loss and increased financial insecurity [3]. At the same time, expenses related to patient care—such as transportation, accommodation near the hospital, medication and subsequent rehabilitation—place a substantial burden on household budgets [4]. This financial strain is particularly severe in countries with insufficient public support or limited welfare structures, creating a “double burden” for caregivers: the emotional weight of uncertainty about the patient’s health and the financial anxiety of sustaining family stability.

Professional repercussions manifest in multiple ways. Working relatives often report reduced productivity, increased absenteeism and difficulties in readjusting after the hospitalization period [5]. In several cases, employers fail to provide the necessary flexibility, resulting in job loss or workplace conflicts. Such changes in professional identity are not restricted to the short term but may exert long-lasting effects on career trajectories, opportunities for advancement and the caregiver’s sense of self-fulfillment [6].

Financial and occupational pressures are closely linked to caregivers’ mental health. Studies consistently report high levels of anxiety, depressive symptoms and post-traumatic stress among relatives of ICU patients [7,8]. The so-called “post-ICU syndrome” does not exclusively affect patients but also extends to their family members, who may experience enduring psychological sequelae. The inability to manage financial strain, combined with the memories of critical illness, contributes to chronic stress, feelings of helplessness and impaired daily functioning [3,9]. Prolonged exposure to these stressors may contribute to mutually reinforcing patterns of financial strain and psychological distress.

Literature also underscores the gendered and social dimensions of these consequences. Women disproportionately shoulder caregiving responsibilities, making them more vulnerable to occupational instability and psychological distress. Likewise, individuals with lower socioeconomic status or insufficient social support networks face heightened risks of long-term psychological and financial consequences [10]. Addressing these inequities is critical for designing targeted interventions and policies that strengthen caregiver resilience.

The present study is conceptually informed by stress–coping and conservation of resources (COR) theoretical frameworks. From a stress–coping perspective, prolonged exposure to critical illness and caregiving demands constitutes a chronic stressor, the psychological impact of which depends on individuals’ appraisal processes and the availability of coping resources. In parallel, conservation of resources theory posits that stress arises from actual or threatened loss of valued resources, including employment, income, role stability, and psychosocial assets. Within this framework, economic and occupational disruptions associated with ICU caregiving represent key resource losses that may precipitate psychological distress, while resilience, coping strategies, and meaning-making processes may buffer—or in some cases exacerbate—these effects depending on context.

The concept of “family resilience” has gained prominence in the context of ICU care. Recent studies suggest that coping strategies, social support and effective communication with the medical team can serve as protective factors against psychological consequences [11,12,13]. Nonetheless, financial and occupational outcomes remain an often-neglected area, despite their strong association with mental health. The development of supportive programs that include financial counseling, legal guidance regarding employment issues and expanded access to mental health services is urgently needed.

Moreover, the COVID-19 pandemic further emphasized the importance of focusing on family caregivers. Increased mortality rates, restricted visitations and social isolation placed relatives under unprecedented strain [14]. Many were forced to grieve without opportunities for closure while simultaneously bearing the financial costs of prolonged hospitalization. These circumstances underscore the need for an interdisciplinary approach that integrates psychological support with social and economic interventions.

In sum, the financial and occupational consequences experienced by relatives of ICU patients represent a multidimensional phenomenon that cannot be disentangled from psychological outcomes. The long-term persistence of psychological sequelae (such as anxiety, depression and post-traumatic stress) is closely associated with financial hardship and occupational instability. Recognizing this interconnection is essential for both clinical practice and health policy. From a research perspective, further investigation is needed into the mechanisms linking financial burden and mental health, as well as evaluations of interventions designed to mitigate these effects at both the individual and systemic levels. Investing in comprehensive support programs not only safeguards caregiver mental health but also strengthens social cohesion and the sustainability of healthcare systems.

Guided by stress–coping and conservation of resources frameworks, the present study had three specific aims. The first is to examine the associations between economic and occupational disruptions (including job loss, reduced working hours, and perceived financial burden) and long-term psychological outcomes among family caregivers of ICU survivors. The second is to investigate how resilience, coping strategies, and spirituality are associated with psychological distress and spiritual engagement at long-term follow-up. The third is to identify distinct caregiver profiles using a person-centered analytic approach, capturing heterogeneity in economic strain, psychological burden, and coping resources.

## 2. Materials and Methods

### 2.1. Study Design and Participants

This study reports a cross-sectional analysis conducted at long-term follow-up within a previously established caregiver cohort. Participants were originally enrolled between 2019 and 2020 during patients’ ICU hospitalization at a tertiary university hospital. The initial cohort included 189 family members of 162 ICU patients. For the present follow-up assessment, conducted approximately five years later, the same participants were re-contacted, and 93 caregivers (mean age 52.2 ± 11.4 years; 60.2% women, 39.8% men) provided complete data. Although participants originated from a longitudinally followed cohort, the present manuscript reports exclusively cross-sectional analyses based on data collected at follow-up. Baseline psychological measures and within-person changes over time were not examined. The follow-up assessment employed different instruments and research questions, focusing on long-term economic, occupational, psychological, and spiritual correlates related to the original ICU experience.

Data collection was carried out through structured questionnaires administered either in-person or via telephone interviews. Standardized psychometric instruments were used to assess psychological symptoms, spirituality, resilience, coping strategies, forgiveness, and personality traits. Additional items captured demographic information, caregiving role, and economic or occupational consequences such as job loss, reduction in working hours, and financial strain.

The follow-up targeted the same cohort of family members who had participated in the initial 2019–2020 study and were caregivers of patients who had survived ICU hospitalization and were discharged from the hospital. Caregivers of patients who died during their ICU stay or before discharge were not included, as post-hospitalization economic and caregiving consequences could not be meaningfully assessed in such cases. Although participants were originally recruited in 2019–2020, the present manuscript reports only cross-sectional analyses of data collected at the follow-up assessment, without longitudinal comparisons to baseline measurements.

All eligible caregivers were recontacted using the telephone numbers provided during the baseline phase. Up to three contact attempts were made for each participant. Of the 189 original caregivers, 93 (49.2%) responded and completed the follow-up assessment.

Of the 189 caregivers initially enrolled during the baseline phase, all were considered eligible for follow-up, provided that the ICU patient had survived to hospital discharge. At follow-up, all 189 caregivers were recontacted using the contact details recorded at baseline. Ninety-three caregivers (49.2%) successfully completed the follow-up assessment. The remaining caregivers were either unreachable after repeated contact attempts (n = 87) or declined participation (n = 9). No statistically significant differences in baseline demographic characteristics were observed between responders and non-responders, as previously reported. Nevertheless, the possibility of selection bias related to differential follow-up cannot be fully excluded.

Inclusion criteria for the follow-up were prior participation in the initial study, age ≥18 years, ability to provide informed consent, and maintenance of caregiving or close familial contact with the ICU survivor.

The study was approved by the Ethics Committee of the University Hospital of Ioannina (protocol code 3263, 1 February 2019), which included the planned follow-up phase, and informed consent was obtained from all participants. The design ensured confidentiality and anonymity, in full accordance with the ethical principles of the Declaration of Helsinki.

### 2.2. Measures

Socio-demographic and caregiving information was collected, including caregivers’ gender, age, relationship to the patient, cohabitation status, number of daily caregiving hours and employment status. Economic and occupational impact variables were assessed through closed-format items examining loss of employment, reduction in working hours, financial burden and perceived career disruption attributable to the caregiving role.

Economic and occupational impact variables were assessed using structured, study-specific items referring to changes attributable to the ICU hospitalization and its aftermath. Job loss was operationalized as a binary variable (yes/no), indicating whether the caregiver had permanently lost employment as a result of caregiving responsibilities following the patient’s ICU admission. Reduced working hours were also coded as a binary variable (yes/no), reflecting a sustained reduction in working time due to caregiving demands.

Perceived financial burden was measured as an ordinal variable using a Likert-type scale ranging from 1 (no financial burden) to 5 (very high financial burden), capturing caregivers’ subjective assessment of economic strain related to the ICU experience and subsequent caregiving period. Career impact was assessed on a similar ordinal scale (1 = no impact to 5 = severe impact), reflecting perceived long-term disruption of professional trajectory, career progression, or employment opportunities. Negative impact on personal and family life was likewise measured on a 5-point Likert scale (1 = no impact to 5 = very strong negative impact).

All economic and occupational items referred to the post-discharge period and the longer-term consequences of the ICU experience, as assessed at the time of follow-up, rather than transient effects during the acute ICU stay.

To evaluate psychological impact, a battery of validated self-report measures was administered. The Symptom Checklist-90-Revised (SCL-90-R) [15] was used to assess psychological distress across multiple dimensions such as somatization, depression, anxiety, hostility, interpersonal sensitivity and psychoticism; the Greek version was applied. Spiritual and religious attitudes were examined using the SpREUK questionnaire [16], which evaluates meaning-making, spiritual trust and reflection; the Greek adaptation was employed [17]. Resilience was measured with the Connor-Davidson Resilience Scale (CD-RISC-10) [18], which captures personal competence, trust in one’s instincts, positive acceptance of change, control and spiritual influences. Dispositional forgiveness was evaluated with the Heartland Forgiveness Scale (HFS) [19], covering forgiveness of self, others and situations. Family coping strategies were measured using the Family Crisis Oriented Personal Evaluation Scales (F-COPES) [20], which assess strategies such as seeking social support, reframing and accepting help.

All instruments were administered in their validated Greek versions and demonstrated satisfactory to high internal consistency in the present sample (Cronbach’s α = 0.79–0.91). All standardized psychometric instruments were used in their validated Greek versions, following appropriate permissions and licensing agreements where required. Permission for the use of the Greek versions was obtained prior to data collection.

### 2.3. Statistical Analysis

Data analysis was conducted using SPSS version 25 (IBM Corp., Armonk, NY, USA) and R version 4.0 (R Foundation for Statistical Computing, Vienna, Austria). Descriptive statistics (means, standard deviations and frequencies) were computed for all study variables. Prior to inferential analyses, data were screened for normality using the Shapiro–Wilk test and inspection of Q–Q plots. Homogeneity of variances was verified with Levene’s test. When assumptions of normality or homogeneity were violated, equivalent non-parametric tests (Mann–Whitney U and Kruskal–Wallis) were applied. Associations between economic and occupational parameters and psychological outcomes were examined through a series of inferential analyses. Specifically, Pearson’s and Spearman’s correlations were employed for continuous variables, while independent-samples *t*-tests and one-way ANOVA were applied for group comparisons across gender, caregiver role and degree of burden. Chi-square tests were used to assess associations between categorical variables. Principal component analysis (PCA) was performed on standardized psychometric variables to identify underlying dimensions of caregiver psychological functioning. Components with eigenvalues greater than 1.0 were retained, and the scree plot was examined to confirm the optimal number of components. Varimax rotation was applied to enhance interpretability. Finally, multivariate linear and logistic regression models were applied to identify predictors of long-term psychological outcomes, focusing particularly on early economic and occupational disruptions as well as caregiving intensity. Principal component analysis (PCA) was employed as a data-reduction technique to address multicollinearity among highly correlated psychometric variables and to identify latent dimensions of psychological distress, coping, and spirituality. These component scores were subsequently used to inform the clustering process, allowing for the identification of caregiver subgroups based on broader multidimensional profiles rather than individual scale scores. This sequential approach was adopted to enhance interpretability and stability of the person-centered analysis.

Prior to interpreting the regression models, key assumptions were tested. Multicollinearity was assessed using variance inflation factors (VIF), with all values below 2 indicating acceptable independence among predictors. All predictors retained in the final regression models exhibited VIF values below commonly accepted thresholds, indicating acceptable levels of collinearity. Nevertheless, given the conceptual and empirical relatedness of several psychometric dimensions, some degree of shared variance among predictors was expected. Homoscedasticity and normal distribution of residuals were verified through visual inspection of scatterplots and Q–Q plots, while standardized residuals were checked for outliers (>±3 SD). When minor deviations from normality were observed, robust standard errors were employed to confirm the stability of the results. A two-tailed *p*-value < 0.05 was considered statistically significant.

In all multivariate regression analyses, the following covariates were entered simultaneously based on theoretical relevance and prior literature: caregivers’ age, gender, relationship to the patient (spouse vs. other), employment status at baseline, and caregiving intensity (hours of caregiving per day), together with the primary economic and occupational predictors (job loss, reduced working hours, perceived financial burden, career impact, and negative impact on personal/family life).

Regression coefficients are presented as unstandardized beta coefficients (β), allowing direct interpretation of the magnitude and direction of the associations between predictors and outcomes.

Missing data were handled using a complete-case analysis approach. Only participants with complete data for the variables included in each specific model were retained, resulting in minor variations in sample size across analyses. Given the exploratory nature of the study and the moderate amount of missing data, no imputation procedures were applied.

To explore potential subgroupings among caregivers based on their psychometric profiles, an initial hierarchical cluster analysis was conducted using Ward’s minimum variance method with squared Euclidean distance as the similarity metric. This approach was chosen because it minimizes within-cluster variance and is suitable for continuous, standardized psychometric data. The two-step cluster analysis was selected due to its suitability for mixed data types and exploratory typology development. Cluster quality was evaluated using internal cohesion and separation criteria generated by the algorithm; however, formal cluster validation indices (e.g., silhouette width) and external validation using independent variables were not performed.

The optimal number of clusters was determined by examining the agglomeration schedule, dendrogram inspection, and changes in the error sum of squares. Based on these criteria, a three-cluster solution was selected and subsequently refined using K-means clustering to maximize within-group homogeneity and between-group differentiation. This two-step procedure (hierarchical followed by K-means) is commonly employed to ensure both data-driven identification and stability of clusters.

Cluster solution quality was evaluated using internal cohesion and separation criteria generated by the two-step algorithm. While formal validation indices such as average silhouette width were not calculated, the stability of the three-cluster solution was supported by clear separation in key psychometric dimensions and consistent interpretability across economic, psychological, and spiritual variables.

## 3. Results

### 3.1. Descriptive Statistics and Correlations

Caregivers reported a broad spectrum of psychological symptoms as assessed by the SCL-90-R, alongside heterogeneous levels of economic and occupational burden. Bivariate analyses using Pearson correlations demonstrated robust associations between specific economic stressors and psychological outcomes. Job loss was positively associated with anxiety and hostility, indicating that occupational disruption correlated with both internalizing and externalizing forms of psychological distress. In contrast, reduced working hours were inversely associated with depression and anxiety. Perceived financial burden, assessed through a single-item subjective indicator, showed weak and non-significant associations with most psychological domains. This pattern should not be interpreted as evidence of a limited role of economic strain but rather as a likely consequence of restricted measurement precision. More concrete and objectively verifiable economic disruptions—such as job loss or sustained reductions in working hours—appeared to function as more sensitive markers of economic stress in this context.

**Figure 1:**
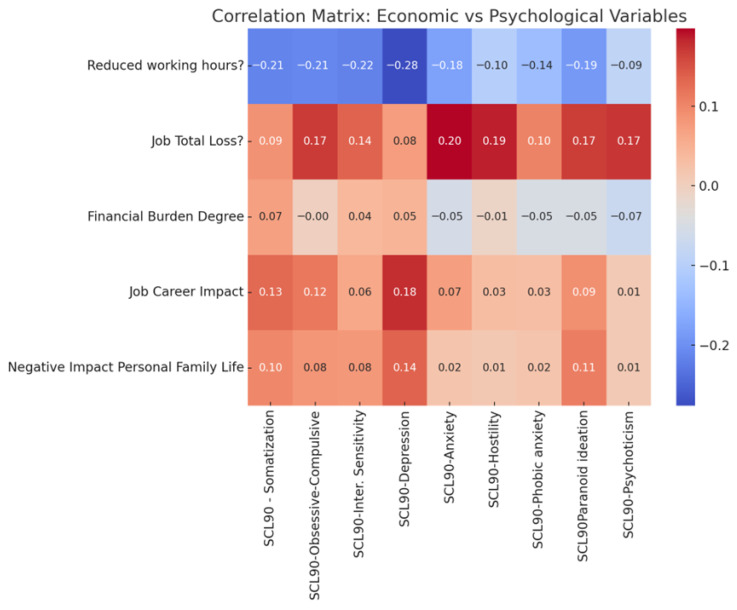
Correlation heatmap illustrating Pearson correlation coefficients between economic and occupational stressors (job loss, reduced working hours, financial burden, career impact) and psychological outcomes measured by SCL-90-R subscales. Color intensity reflects the strength and direction of correlations, with darker shades indicating stronger positive or negative associations.

A correlation heatmap (Figure 1) was generated to visually represent the associations between economic/occupational stressors and psychological outcomes (SCL-90-R subscales). The intensity of the color denotes the strength and direction of the Pearson correlation coefficients, with darker shades indicating stronger associations. The heatmap highlights the strong positive associations of job loss with anxiety and hostility, the inverse associations of reduced working hours with depression and anxiety and the generally weak correlations between perceived financial burden and psychological symptoms.

### 3.2. Regression Models for Psychological Outcomes

Table 1, Table 2 and Table 3 provide a comprehensive overview of the multivariate regression and clustering analyses. Table 1 presents standardized regression coefficients (β) and *p*-values for the prediction of psychological symptoms from economic and occupational variables among ICU caregivers, with significant predictors (*p* < 0.05) highlighted in bold. Table 2 reports the mean scores of economic and occupational impact variables across the three caregiver clusters identified through K-Means clustering on psychometric profiles, illustrating the differential burden across subgroups. Table 3 summarizes significant predictors (*p* < 0.05) of spirituality-related outcomes (SpREUK total score, subscales and CD-RISC-10 Spiritual Influences), showing the combined effects of psychopathology, coping strategies, forgiveness, personality traits, quality of life, and economic stressors.

The cluster analysis identified three distinct caregiver profiles reflecting relative patterns across economic, psychological, and coping-related dimensions. Cluster 0 was characterized by high overall burden, with prominent psychological distress and substantial economic and occupational strain accompanied by limited coping resources. Cluster 1 represented an intermediate profile, marked by moderate psychological symptoms and a mixed pattern of coping and spiritual engagement. Cluster 2 reflected a resilient profile, characterized by low psychological distress, comparatively fewer economic disruptions, and stronger resilience and adaptive coping capacities.

It is also important to note that while several CD-RISC-10 subscales were statistically associated with spirituality outcomes, these findings should be interpreted cautiously due to strong intercorrelations among the subscales. Several CD-RISC-10 subscales were statistically associated with spirituality outcomes, but due to strong intercorrelations among the subscales, only the total CD-RISC-10 score was retained as the most consistent predictor of spiritual coping.

Overall, job loss was positively associated with multiple psychopathology dimensions, reaching statistical significance for anxiety and hostility, while reduced working hours were inversely associated with depression. The association of spirituality indicators with psychological variables reflected a complex pattern, whereby higher spirituality scores tended to co-occur with elevated distress levels, suggesting a reactive pattern of coping.

PCA revealed three principal components with eigenvalues greater than 1.0, jointly explaining 68.91% of the total variance. The first component reflected generalized psychological distress (high loadings on depression, anxiety, and hostility), the second captured spiritual coping and meaning-making, and the third represented resilience and adaptive functioning.

**Figure 2:**
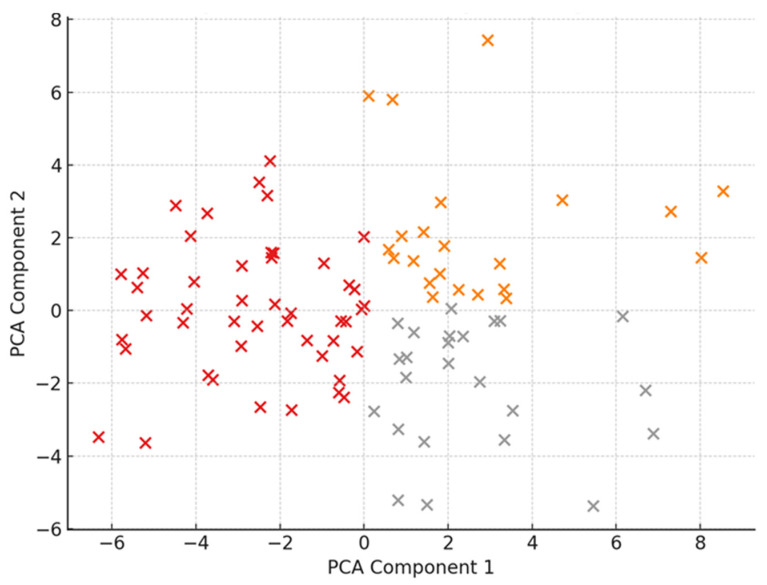
Cluster analysis of caregiver psychometric profiles based on principal component scores, illustrating three distinct caregiver groups characterized by differing levels of psychological distress, economic burden, and spiritual coping.

Figure 2 complements these findings by depicting the three distinct caregiver clusters derived from PCA: one characterized by high psychological burden coupled with severe economic strain, another reflecting moderate distress and mixed reliance on spiritual coping, and a third demonstrating low psychopathology and minimal economic burden. These clustering patterns indicate heterogeneity among caregivers, with economic disruptions closely related to psychological symptoms and spirituality emerging as a coping mechanism predominantly under conditions of elevated distress.

## 4. Discussion

This study examined the complex interplay between economic burden, psychological distress and spiritual coping among family caregivers of ICU patients, employing an extensive psychometric assessment and cluster analytic approach. The findings provide novel insights into the lived experiences of caregivers while at the same time confirming trends observed in previous research and pointing to underexplored dimensions in the current literature.

Economic and occupational stressors emerged as particularly strong factors associated with psychological outcomes. In line with earlier work [21,22], job loss was one of the strongest predictors of anxiety and hostility, whereas reduced working hours were inversely associated with depressive symptoms. This association should not be interpreted as uniformly protective but rather as context-dependent. These results underscore that objectively defined occupational disruptions, particularly job loss, are centrally associated with caregiver psychological burden. In contrast, perceived financial burden showed weaker and less consistent associations with psychopathology, a finding that should be interpreted cautiously in light of measurement limitations. The use of a single-item, subjective indicator of financial strain may have reduced sensitivity to the full scope of economic hardship experienced by caregivers, potentially attenuating observed associations. Recent evidence from the COVID-19 era reinforces this, showing that informal caregivers face substantial out-of-pocket expenses, loss of income and worsened mental health outcomes when caregiving responsibilities intersect with economic instability [23].

Within the Greek context in particular, where formal caregiver support policies, paid family leave, and comprehensive income protection mechanisms remain limited, reduced working hours may reflect heterogeneous and even opposing processes. For some caregivers, reduced hours may indicate access to flexible employment arrangements or supportive family resources; for others, they may represent constrained labor participation, income loss, and heightened financial risk. Consequently, the observed inverse association with depressive symptoms should be interpreted cautiously and cannot be generalized beyond socio-economic contexts with comparable labor and welfare structures.

Although the study employed a longitudinal follow-up design, the present analyses are based on cross-sectional data collected at the five-year assessment. Consequently, observed associations should not be interpreted as reflecting specific causal pathways. For example, the inverse association between reduced working hours and depressive symptoms may be interpreted in multiple, potentially opposing ways. Reduced working hours may function as a protective coping strategy that facilitates psychological adjustment; alternatively, it may reflect socioeconomic privilege and access to flexible employment conditions; or it may represent a consequence of underlying psychological distress that limits full-time work capacity. In the absence of baseline psychological measures and repeated assessments of employment status analyzed longitudinally, the temporal ordering of these processes cannot be determined. The findings therefore highlight patterns of co-occurrence rather than directional effects.

Importantly, the economic and psychological burdens observed in this study should not be interpreted solely as outcomes of individual coping capacities, personality traits, or spiritual orientations. Rather, they are embedded within broader structural and policy contexts that shape caregivers’ exposure to risk and available resources. The “double burden” of caregiving and economic strain is not a natural or inevitable phenomenon but one that is produced and amplified by institutional arrangements and social policy choices. In the absence of paid caregiver leave, limited income replacement mechanisms, weak social safety nets, and fragmented access to mental health services, caregivers are often compelled to absorb the economic and emotional costs of critical illness privately. Gender norms further exacerbate this burden, as caregiving responsibilities disproportionately fall on women, increasing their risk of employment disruption, financial insecurity, and long-term psychological distress. Within this context, individual-level factors such as coping strategies or spirituality may shape subjective experience, but they operate within—and cannot compensate for—structural constraints. From this perspective, the observed associations between economic hardship and psychological outcomes reflect not only personal vulnerability but also systemic failures to provide adequate institutional support for informal caregivers. Addressing caregiver burden therefore requires not only psychosocial interventions but also policy-level responses targeting labor protections, caregiver leave provisions, and equitable access to mental health care.

Spirituality also played an important, though nuanced, role. Spirituality-related measures (SpREUK; CD-RISC-10 Spiritual Influences) were significantly associated with outcomes, yet the pattern of results suggests that spirituality is often activated reactively under distress rather than serving consistently as a primary protective factor. This aligns with qualitative findings of caregivers invoking spiritual beliefs and practices in ICU settings when experiencing high stress and uncertainty.

An especially noteworthy finding of this study is the way in which spirituality intersects with economic hardship and depressive symptomatology. Caregivers who reported higher financial strain and job-related disruptions tended to exhibit elevated scores in spiritual coping dimensions, suggesting that spirituality was mobilized as a compensatory mechanism in contexts of material and emotional deprivation. This pattern resonates with recent evidence that economic insecurity amplifies existential concerns, thereby intensifying reliance on spiritual or religious frameworks for meaning-making [24]. At the same time, our regression models revealed that certain facets of spirituality, particularly reliance on external spiritual support, were positively associated with depression rather than mitigating it. This paradox highlights the dual role of spirituality: while it may offer short-term relief through hope and transcendence, it may also reflect an underlying sense of helplessness when practical economic support is lacking. In this light, spirituality should not be regarded as uniformly protective; rather, it interacts with socioeconomic vulnerabilities and psychological states, sometimes reinforcing distress. Accordingly, the regression findings should be viewed as indicative of broader relational patterns among psychological, economic, and spiritual dimensions rather than as precise estimates of independent effects for highly interrelated constructs. These findings strengthen the argument for integrated interventions that simultaneously address financial insecurity and provide structured, proactive spiritual care, in order to prevent spirituality from becoming a marker of despair instead of a source of resilience.

The interpretation of spirituality-related findings, particularly those derived from the SpREUK dimensions of “search for meaning” and “reflection,” warrants careful qualification. Although these dimensions are often conceptualized as indicators of adaptive meaning-making, heightened engagement in spiritual searching during periods of crisis may also reflect unresolved existential distress or ruminative cognitive processes. In such contexts, elevated scores may capture a state of ongoing struggle rather than successful integration or spiritual growth.

Accordingly, the observed positive associations between spiritual searching and depressive symptoms should not be interpreted as evidence that spirituality per se exacerbates psychological distress. Rather, they may reflect partial conceptual overlap between spiritual rumination and psychopathological rumination, a distinction that may not be fully disentangled by self-report instruments such as the SpREUK. This complexity underscores the need to interpret spirituality-related findings as dynamic and context-dependent processes rather than uniformly protective or maladaptive constructs.

The ambivalent role of spirituality observed in this study may be further understood through the distinction between positive and negative religious coping. Heightened spiritual engagement following critical illness may reflect not only adaptive meaning-making but also forms of spiritual struggle, such as feelings of abandonment, perceived punishment, or existential doubt. In this sense, the observed association between reactive spirituality and depressive symptoms may be indicative of negative religious coping rather than a protective spiritual resource. Self-report instruments such as the SpREUK capture dimensions of spiritual searching and reflection, but they may not fully differentiate between constructive spiritual integration and distress-driven rumination or spiritual struggle. This measurement overlap may partly explain why increased spiritual engagement co-occurred with higher depressive symptomatology in the present sample. Future research would benefit from incorporating instruments specifically designed to distinguish positive from negative religious coping, such as the Brief RCOPE, as well as longitudinal assessments, to examine how spiritual coping trajectories evolve over time following ICU discharge. The finding that spirituality may function as both a resource and a marker of distress has important implications for clinical practice. Proactive spiritual care should not be understood as the promotion of religious beliefs but as the provision of non-imposing, culturally sensitive support that acknowledges patients’ and caregivers’ existential concerns. In the ICU context, such care may include routine screening for spiritual distress, access to trained chaplains or spiritual care professionals, and integration of spiritual assessment into interdisciplinary care planning. Crucially, spiritually integrated care should respect pluralism and individual preferences, offering support without presupposing religious affiliation or normative spiritual frameworks. When appropriately embedded within clinical practice, spiritual care may help identify caregivers at risk of unresolved existential distress rather than serving as a universal coping intervention.

Beyond economic and spiritual dimensions, individual traits and coping strategies were also influential. High neuroticism consistently predicted worse psychological outcomes, while adaptive coping mechanisms—such as cognitive reframing, forgiveness and personal competence—aligned with lower anxiety and better resilience. These observations are consistent with emerging studies that highlight personality traits and adaptive coping as key moderators of caregiver burden [24].

The cluster analysis further underscored the heterogeneity of caregiver experiences. Three distinct groups emerged: a high-burden cluster characterized by elevated psychopathology and severe economic strain; a moderate cluster with mixed psychological and spiritual profiles; and a resilient cluster marked by low psychological distress and minimal economic burden. This typology demonstrates that caregivers cannot be regarded as a homogeneous population. Interventions must be tailored: some caregivers may primarily need economic and occupational support, others may benefit more from psychological counseling or structured spiritual care and yet others may be supported by resilience-enhancing programs.

The cluster analysis revealed three distinct caregiver profiles, each carrying specific psychological meanings. The high-burden cluster, characterized by elevated psychopathology and severe economic strain, can be understood as reflecting caregivers who are overwhelmed by cumulative stressors and unable to mobilize adequate coping resources. This group exemplifies the risk of caregiver burnout and illustrates how economic insecurity exacerbates vulnerability to anxiety, depression and hostility, consistent with meta-analytic evidence that caregiving is associated with significant health risks when stressors accumulate [25].

The moderate cluster, with intermediate levels of psychological distress and spirituality, appears to represent a transitional group in which caregivers oscillate between adaptive and maladaptive strategies. Their reliance on spirituality alongside moderate psychological symptoms suggests an ongoing struggle to find balance between external resources and internal resilience. This dynamic resonates with research emphasizing the complex psychological toll of family caregiving and the mixed trajectories that often emerge [8]. Finally, the resilient cluster, marked by low psychopathology and minimal economic burden, demonstrates the protective role of personal competence, effective coping mechanisms and supportive environments. This group reflects what psychological models of stress and coping describe as successful appraisal and resource mobilization in the face of adversity [26]. Taken together, these clusters highlight the heterogeneity of caregivers and reinforce the importance of personalized interventions that address not only economic conditions but also individual differences in coping, resilience and meaning-making. Accordingly, the identified caregiver clusters are best viewed as heuristic profiles that capture meaningful patterns of co-occurring burden rather than as fixed or fully validated caregiver subtypes.

Beyond the empirical findings of the present study, psychoanalytic theory has historically offered conceptual frameworks for understanding experiences of loss, dependency, and existential threat in caregiving contexts. The strong association between economic loss and heightened anxiety or hostility may be read as a displacement of feelings of helplessness and fear of loss of the loved one onto the tangible domain of financial insecurity. In psychoanalytic terms, the economic disruption becomes a concrete representation of symbolic castration and loss, intensifying depressive and anxious symptomatology [27]. Similarly, the observation that spirituality often emerges reactively and is linked in some cases with higher depression may signify the ambivalent role of faith as both a container of despair and a regression to infantile reliance on an omnipotent other [28]. Depression in caregivers, particularly when coupled with high spiritual reliance, could thus be viewed as an expression of unresolved mourning processes and the collapse of ego defenses in the face of cumulative losses [29]. This psychoanalytic lens highlights how caregiving in the ICU context is not only a social and psychological burden but also a profound confrontation with dependency, mortality and existential vulnerability.

Taken together, these results move beyond merely replicating earlier findings; they quantify the relative contributions of economic, psychological and spiritual factors in multivariate contexts, thus advancing the caregiver burden literature. Importantly, they confirm that ICU caregiving entails not only psychological challenges but substantial socioeconomic consequences and that the pathways through which caregivers adjust are multidimensional. Spirituality and coping resources emerge as reactive, yet potentially transformative, mechanisms that may buffer distress when mobilized effectively. Overall, these findings reinforce that ICU caregiving constitutes a multidimensional burden, encompassing emotional, socioeconomic and existential domains. Addressing caregiver well-being requires integrated approaches that span healthcare, social and occupational systems. Ultimately, addressing caregiver burden in ICU settings requires a paradigm shift from patient-centered to family-centered intensive care, where economic, psychological and spiritual needs are considered inseparable dimensions of recovery.

The present findings should therefore be interpreted as describing the long-term consequences of ICU caregiving in the context of patient survival and ongoing post-discharge adaptation, rather than the full range of caregiving outcomes following critical illness.

### 4.1. Implications and Recommendations

The findings of this study carry several important implications for clinical practice, policy and future research. At the clinical level, the results highlight the potential relevance of economic and occupational support for ICU caregivers, including job protection, flexible work arrangements, and access to financial counseling. These factors were consistently associated with psychological burden in the present analyses, indicating that economic strain may represent an important contextual correlate of caregiver distress rather than a peripheral concern. Beyond economic aspects, the findings also point to the complex role of spirituality, suggesting that spiritual care, when delivered in a sensitive and non-imposing manner, may represent a relevant area for clinical attention. Approaches such as the systematic assessment of spiritual needs and the facilitation of meaning-oriented conversations could be considered as supportive resources, particularly for caregivers experiencing elevated psychological distress. In parallel, interventions aimed at strengthening adaptive coping strategies—including cognitive reframing, forgiveness, and personal competence—may be relevant avenues for enhancing caregiver resilience.

Future research should move beyond reliance on single-item, subjective indicators of financial strain and incorporate objective, quantifiable economic measures alongside perceived burden. The use of validated instruments assessing family financial burden, as well as direct indicators such as income loss, accumulated debt, out-of-pocket medical and caregiving-related expenses, and depletion of savings, would allow for a more precise and comprehensive estimation of the economic consequences of ICU caregiving. Combining objective economic metrics with subjective appraisals may also help clarify the pathways linking financial strain and psychological outcomes.

From a research perspective, the study underscores the need for longitudinal, multicenter designs to clarify temporal relationships between economic hardship, psychological symptoms and spiritual coping. Future work should also employ advanced analytic methods such as structural equation modeling (SEM) or penalized regression techniques (e.g., LASSO, Elastic Net) to address multicollinearity and disentangle the overlapping influences of multiple psychosocial dimensions. Collectively, these steps will contribute to a more nuanced understanding of caregiver burden and inform targeted, multidimensional interventions.

### 4.2. Limitations

Although the study involved a five-year follow-up, the primary inferential analyses were cross-sectional and conducted at the follow-up assessment, which limits causal inference. The temporal ordering between economic hardship and psychological distress cannot be established, and reverse or bidirectional pathways remain possible. Baseline psychological status and employment characteristics were not modeled longitudinally, preventing distinction between alternative trajectories (e.g., whether economic changes preceded or resulted from psychological distress). Accordingly, findings should be interpreted as cross-sectional associations observed at long-term follow-up rather than evidence of directional effects.

Missing data across some psychometric measures reduced the effective sample size in certain models, potentially limiting statistical power and generalizability. In addition, reliance on self-report instruments for psychological symptoms, spiritual dimensions, and perceived financial burden introduces the possibility of recall bias and social desirability effects. Economic burden was assessed primarily through single-item subjective indicators, without objective economic measures such as income loss, debt accumulation, or out-of-pocket expenses. This may have attenuated associations with psychological outcomes, rendering estimates of financial impact conservative; in contrast, more concrete events (e.g., job loss or reduced working hours) showed more robust associations, likely reflecting greater measurement reliability.

Attrition over the long follow-up period represents a further limitation. Although baseline demographic characteristics did not differ significantly between responders and non-responders, selective loss to follow-up cannot be excluded. Caregivers experiencing the highest levels of psychological distress or socioeconomic instability may have been underrepresented, potentially leading to underestimation of long-term burden. This limitation is particularly relevant for the cluster analysis, as the size and characteristics of the high-burden cluster may be conservative.

The cluster solution itself should be interpreted as exploratory. While the two-step clustering approach is appropriate for mixed-variable datasets, formal validation indices (e.g., silhouette width) and external validation using independent variables were not performed. Consequently, the identified clusters cannot be considered stable or clinically definitive subgroups.

An important conceptual limitation is the exclusion of caregivers of patients who died during the ICU stay. Bereavement following ICU death is associated with distinct and often more severe psychological, spiritual, and economic consequences. By focusing exclusively on caregivers of ICU survivors, the study captures only one caregiving trajectory and does not reflect the full spectrum of ICU-related caregiver burden. This deliberate methodological choice enhances conceptual homogeneity but limits generalizability and completeness of the conclusions.

Finally, the findings are most applicable to caregivers within a single-payer European healthcare context, where labor protections and social welfare systems may buffer economic and psychological strain. Generalization to settings with fragmented healthcare financing or weaker social safety nets should therefore be made with caution. Psychoanalytic interpretations referenced in the discussion remain theoretical, as the study relied exclusively on psychometric self-report data and did not include qualitative or clinical assessments capable of supporting inferences about unconscious or symbolic processes.

## 5. Conclusions

In conclusion, this study highlights the multifaceted burden experienced by family caregivers of ICU patients, demonstrating that economic stressors—particularly job loss and reduced working hours—are strongly associated with psychological distress. At the same time, spirituality frequently emerged as a resource mobilized in response to existing strain rather than as a primary protective factor. Importantly, personal resources such as coping strategies, forgiveness, resilience and personality traits modulated the degree of psychological vulnerability, underscoring the heterogeneity among caregivers. By integrating economic, psychological and spiritual dimensions, these findings emphasize the need for multidimensional, tailored interventions that address not only the emotional but also the socioeconomic realities of caregiving. Such an approach has the potential to reduce long-term psychological sequelae, enhance resilience and ultimately improve both caregiver well-being and patient care.

## Figures and Tables

**Table 1:** Summary of multiple linear regression models examining associations between economic and occupational variables and psychological outcomes among ICU caregivers. Values represent unstandardized beta coefficients (β) and corresponding *p*-values.

Dependent Variable	Reduced Working Hours (β, *p*)	Job Loss (β, *p*)	Financial Burden (β, *p*)	Career Impact (β, *p*)	Personal/Family Impact (β, *p*)
Depression	−4.43, 0.01	4.52, 0.18	−0.64, 0.456	0.39, 0.62	0.42, 0.61
Anxiety	−3.01, 0.03	5.91, 0.02	−0.42, 0.522	0.17, 0.78	0.03, 0.95
Obsessive-Compulsive	−3.44, 0.02	6.12, 0.03	−0.35, 0.534	0.01, 0.98	0.22, 0.68
Somatization	−3.58, 0.04	3.87, 0.16	−0.23, 0.721	0.30, 0.63	−0.08, 0.90
Hostility	−0.37, 0.61	2.85, 0.03	−0.34, 0.505	0.26, 0.64	0.19, 0.70

**Table 2:** Conceptual profiles of caregiver clusters derived from multivariate psychological, economic, and coping indicators.

Cluster	Reduced Working Hours	Job Loss	Financial Burden Degree	Job Career Impact	Negative Impact Personal/Family Life
Cluster 0	1.44	2.00	3.38	2.72	3.22
Cluster 1	1.57	1.94	3.51	2.49	2.97
Cluster 2	1.62	1.85	3.50	2.73	3.19

**Table 3:** Summary of multivariate regression models examining predictors of spirituality-related outcomes (SpREUK subscales and CD-RISC-10 Spiritual Influences). Reported values represent unstandardized beta coefficients (β) and *p*-values.

Outcome	Predictor	Beta	*p*-Value
SpREUK Total Score	Total CD-RISC-10	1.36	0.00
SpREUK Trust in Higher	Total CD-RISC-10	0.72	0.00
SpREUK Reflection	Total CD-RISC-10	0.37	0.00
SpREUK Reflection	Eysenck—Neuroticism	0.59	0.01
CD-RISC-10—Spiritual Influences	Total Self-Compassion	0.00	0.01
CD-RISC-10—Spiritual Influences	CD-RISC-10—Personal Competence	−1.00	0.00
CD-RISC-10—Spiritual Influences	CD-RISC-10—Trust in Instincts	−1.00	0.00
CD-RISC-10—Spiritual Influences	CD-RISC-10—Positive Acceptance	−1.00	0.00
CD-RISC-10—Spiritual Influences	CD-RISC-10—Control	−1.00	0.00
CD-RISC-10—Spiritual Influences	Total CD-RISC-10	5.00	0.00
CD-RISC-10—Spiritual Influences	SCL90—Anxiety	0.00	0.03

Note: All coefficients are derived from multivariable regression models adjusted for relevant covariates. Individual predictors are presented separately for clarity, but estimates reflect their effects within multivariable models rather than simple bivariate associations.

## Data Availability

The data presented in this study are available on request from the corresponding author. The data are not publicly available due to privacy and ethical restrictions.

## Abbreviations

The following abbreviations are used in this manuscript:

ICU	Intensive Care Unit
SD	standard deviation
PCA	Principal Component Analysis
VIF	Variance Inflation Factors
SEM	Structural Equation Modeling
